# Non-Markovianity hinders Quantum Darwinism

**DOI:** 10.1038/srep19607

**Published:** 2016-01-20

**Authors:** Fernando Galve, Roberta Zambrini, Sabrina Maniscalco

**Affiliations:** 1 IFISC (UIB-CSIC), Institute of Cross-Disciplinary Physics and Complex Systems, UIB Campus, 07122 Palma de Mallorca, Spain; 2 Turku Center for Quantum Physics, Department of Physics and Astronomy, University of Turku, FIN-20014 Turku, Finland

## Abstract

We investigate Quantum Darwinism and the emergence of a classical world from the quantum one in connection with the spectral properties of the environment. We use a microscopic model of quantum environment in which, by changing a simple system parameter, we can modify the information back flow from environment into the system, and therefore its non-Markovian character. We show that the presence of memory effects hinders the emergence of classical objective reality, linking these two apparently unrelated concepts via a unique dynamical feature related to decoherence factors.

Quantum Darwinism is a fascinating theory that explains the emergence of a classical objective reality in terms of proliferation of information about certain states of a quantum system into the environment^1^,^2^. We live in a quantum Universe, the behaviour of all microscopic constituents being described by the laws of quantum physics. There is overwhelming evidence that this incredibly successful theory applies at all scales. Why then the macroscopic objects populating our everyday reality are only found in a much smaller subset of states, consistent with classical laws?

The emergence of the classical objective world from the quantum one has been debated and investigated since over a century^3^. A significant advance in our understanding of the quantum measurement problem was achieved through the theory of environment induced decoherence^4^, showing how continuous monitoring by the environment destroys fragile quantum information and allows only certain classical states to survive^5^. Quantum Darwinism takes a further step promoting the role of the environment from a passive sink of coherence to the active carrier of information about the system. Indeed, as Zurek observes^1^, generally we do not measure quantum systems directly, rather we infer their properties by the observation of parts of the environment. The question addressed by quantum Darwinism is whether, and which, information about the system is redundantly imprinted in independent, and thus uncorrelated, fractions of the environment. Such fractions can then be measured by many independent observers that will detect the same property of the system, without perturbing it. This illustrates the emergence of an objective classical reality from the quantum probabilistic world. The environment of a quantum system can be imagined as an almost endless collection of independent degrees of freedom where information can be massively and redundantly stored through interaction with the system. Which kind of information is spread and under which conditions? This is the subject of Quantum Darwinism. From the no-cloning^6^ theorem, we can expect that the full quantum information of the state of the system is not copied into the environment, and thus only partial (classical) information is spread. Further, it is but natural that the environment-amplified information is related to the observable which couples the system with its surroundings, usually called pointer observable^5^.

A recent breakthrough in the field^2^ has shown that spreading of the classical information about a pointer observable into the environment is a generic feature of quantum mechanics. They show that a generic quantum evolution of system plus a *large* environment results in a measure and prepare map for fractions in the environment, that is, only information about measurement outcomes of a particular POVM are scattered around and thus proliferate. Fractions can be either independent degrees of freedom, or collections thereof. However, the imprints of such results, which give information about the system’s pointer observable corresponding to that POVM, can be non-informative: each outcome can be encoded in non-orthogonal supports, and thus they cannot be distinguished from observation of that environment fraction. Thus, it is not only a matter of spreading copies around, these copies have to be informative. As demonstrated in^7^, a randomly picked dynamics, will produce a rather non-informative scattering of information: only chunks of half the environment’s size will convey enogh information about the system. Therefore, fulfilment of Quantum Darwinism, in which also very small fragments of the environment are fully informative, is a peculiarity of the physical interactions that we have in nature.

This latter peculiarity, that close to full information about the system pointer is *redundantly* copied among many small fragments in the surroundings, has been recently shown for all models of pure decoherence^8^, for the photonic environment^9^,^10^,^11^, and in general for a two level system under a (environment symmetric) controlled unitary evolution for the bath^12^. Experimental evidence for its workings in a quantum dot scenario has been given in^13^.

The main result of this article is the discovery of a strong connection between the non-Markovian character of the open quantum system dynamics, indicated by the presence of information flowback^14^, and the inhibition of such redundancy and hence objectivity. In the non-Markovian dynamics setting^14^,^15^,^16^,^17^,^18^,^19^,^20^,^21^,^22^,^23^,^24^,^25^, indeed, measurements by the observers would inevitably subtract part of the information present in the environment that could feedback into the system, hence perturbing the system’s subsequent state.

## Results

We illustrate this effect by looking at a paradigmatic model, the quantum Brownian oscillator. This model has been extensively studied in the literature (see, e.g., ref. 5) to describe vibrational/bosonic modes dissipating into bosonic reservoirs. Its behaviour in terms of quantum Darwinism has been studied in the case of Markovian evolution^26^ and in the more generic scenario of super-Ohmic bath spectrum^27^. The non-Markovian properties of this model for non-Ohmic spectral densities were explored recently in^28^, where the relation between resonant/detuned situations and non-Markovianity was pinpointed. We here fill the gap and show that non-Markovian behaviour leads to breaking of the process of quantum Darwinism, thus hindering the production of stable records in the bath.

The model consists of a quantum harmonic oscillator of frequency *ω*_*S*_ coupled through the bilinear interaction 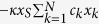 to a quantum environment of *N* harmonic oscillators having frequencies *ω*_*k*_ = *ω*_0_ + *k*Δ, with Δ = (*ω*_*R*_ − *ω*_0_)/*N*, and *x*_*S*_ and *x*_*k*_ position operators of system and environment oscillators, respectively. The spectral density is given by


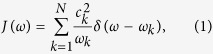


which becomes a function in the continuum limit. In typical scenarios an Ohmic (*s* = 1) frequency dependence 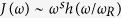 with *h*(*ω*/*ω*_*R*_) a frequency cutoff function which decays fast for *ω* > *ω*_*R*_ is used, with most variations covering the *s* ≠ 1 cases. A possible microscopic derivation of such Ohmic dissipation is based on the Rubin model^29^, consisting of a homogeneous linear harmonically coupled chain of equal on-site frequency (*ω*_0_) oscillators with coupling strength *g*, leading to a spectral density 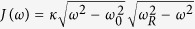 with 
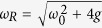
. This microscopic model reproduces an Ohmic spectral density for *ω*_0_ = 0, while it allows for the study of non-Markovian dynamics when the system is detuned with respect to such bath.

### Classical records of the quantum Brownian particle

We consider the case in which the system oscillator is initially prepared in a momentum-squeezed state with squeezing parameter *r* and the environment is in the vacuum state. Because the system and bath couple through position, this is going to be the classically ‘recorded’ observable of the system, meaning that the initial position spread will be redundantly stored in fragments of the environment. In this sense we expect, if perfect quantum Darwinism occurs, the initial state of the system


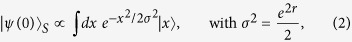


to be perfectly broadcast^2^,^11^ into the environment


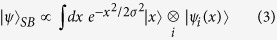


as independent, perfectly distinguishable branches 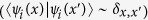 which are conditional on each initial position. Notice that the latter condition ensures a perfect correlation, and thus knowledge, between the probability to obtain a given 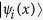 for bath’s oscillator *i* and the position of the system; and not only for one oscillator, but for any collection of them. In this sense, the information on the position observable of the system gets redundantly recorded into the environment.

Dynamically, reaching such kind of state can be seen by using the state analysis in^26^ and^27^, where the Brownian oscillator is considered very massive and thus underdamped. In such approximation, backreaction of the environment on the system is very small, and system mediated interactions between bath modes are negligible. Hence the bath oscillators suffer an evolution conditioned upon the value of the system’s position, as given by equation (3).

It is easy to show that in this ideal situation the evolution matches a ‘measure and prepare’ map^2^ (which measures the system and prepares an orthogonal recording of its outcomes on each bath mode) for system + fraction and furthermore that such branching state can be written in ‘spectrum broadcast’ form^11^


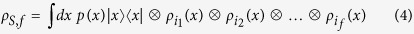



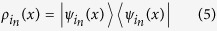


with 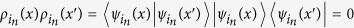 for *x* ≠ *x*′, and the fraction is composed by oscillators 
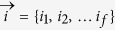
.

Of course, the perfect distinguishability of records 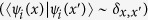 is the ideal situation, and it can be shown^26^ that 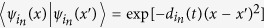, where 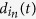 is a decohering factor highlighting the strength with which fraction *i*_*n*_ of the bath is able to decohere the system. This means that perfect Darwinism is reached when such factor is big enough. Intuitively, if this ideal situation is achieved, each fragment of the environment can perfectly distinguish the different classical position records of the system, and just by interrogating a fraction (*any* fraction, of *any* size) we can gain the information on the label *x*.

### Non-Markovianity

Our main goal is to discuss the connection between non-Markovianity and the dynamical onset of objectification. We will therefore use and compare the non-Markovianity measure for continuous variables systems introduced in ref. 30 on the one hand, and the mutual information between system and fractions of the environment^26^ on the other hand.

We detect and quantify the presence of memory effects in terms of information flow back as indicated by the non-monotonic behaviour of fidelity between pairs of states *F*(*ρ*_1_, *ρ*_2_). Fidelity monotonically increases under the action of completely positive and trace preserving maps, indicating a decrease in state distinguishability and hence a loss of information on the system due to the action of the environment. A temporary decrease of fidelity for certain time intervals therefore signals a partial increase of information on the system, i.e., information flow back. The corresponding measure of non-Markovianity is obtained by optimizing over all pairs of states^30^


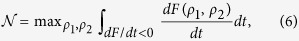


where the integral extends over all time intervals in which the derivative of the fidelity is negative. In practice, we restrict our attention to Gaussian states, and calculate the corresponding Gaussian measure of non-Markovianity.

The open quantum system model here considered has the property that, by varying the frequency of the system oscillator with respect to the spectral distribution, we change the non-Markovian character of the open system dynamics^28^. Specifically, higher values of non-Markovianity are obtained close to the edges of the spectral distribution while on resonance non-Markovianity is minimal, as we further illustrate in the following.

### Redundant storage of information

In describing quantum Darwinism the key quantity of interest describes correlations between the system and fragments of the environment of size *f*. These correlations are given by the mutual information


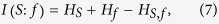


with *H*_*S*_ and *H*_*f*_ the individual von Neumann entropies of system and environmental fraction, and *H*_*S*,*f*_ the entropy of the combined system. If the initial state is pure the quantity *H*_*S*_ is the entropy of the system due to decoherence and it therefore measures all the information about *S* available from either the system or the environment. The emergence of an objective reality through decoherence is indicated by the existence of a very horizontal plateau around *f* = *N*/2 in the plot of 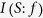 as a function of *f*. More precisely the values of 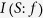 quickly raise to *H*_*S*_, indicating that already small factions of the environment contain almost all information classically accessible on the system. A typical indicator^1^ is the redundancy *R*_*δ*_ = 1/*f*_*δ*_, with *f*_*δ*_ the fraction size which already supplies almost all classical information on the system, i.e. (1 − *δ*)*H*_*S*_. Large redundancy, or equivalently small values of *f*_*δ*_, show that the state of the system can be found independently by many observers (probing e.g. independent fractions of different sizes), who agree on the outcome, by measuring parts of the environment without disturbing the system. An important point is also the fact that an observer needs not collect information on a huge set of environmental degrees of freedom, impossible in practical terms, in order to get an idea of the state of the system.

**Figure 1:**
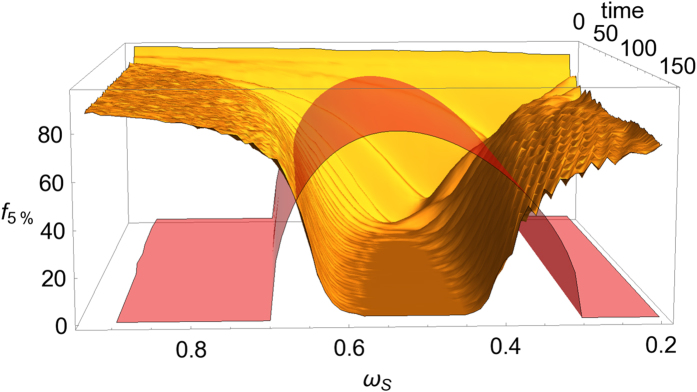
Behaviour of *f*_*δ*_ = 1/*R*_*δ*_ for *δ* = 5% as a function of time (a.u.) and of the system frequency *ω*_*S*_ for a bath of *N* = 300 oscillators with central frequency *ω*_0_ = 0.3 and cutoff frequency *ω*_*R*_ = 0.7. The initial squeezing parameter is *r* = 10. We have artificially drawn (transparent red) the spectral density *J*(*ω*_*S*_) along time for illustrative purposes.

**Figure 2:**
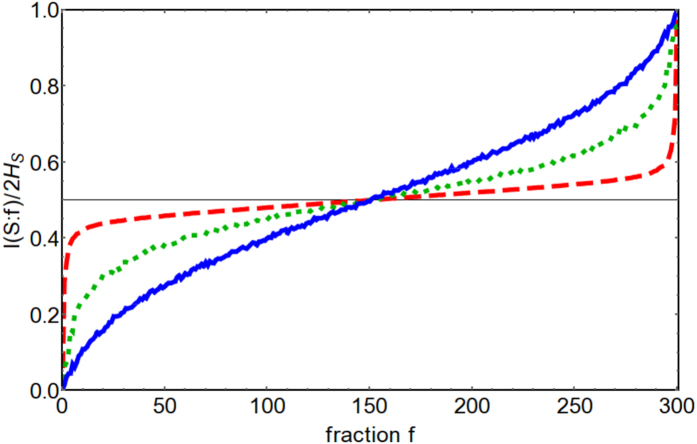
Mutual Information 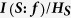 as a function of the fraction *f* for *t* = 40 (a.u.) and *ω*_*S*_ = 0.5 (red/dashed line), *ω*_*S*_ = 0.7 (green/dotted line) and *ω*_*S*_ = 1 (blue/solid line), with *r* = 3. All other parameters are the same as in Fig. 1.

We choose randomly fractions of the environment and investigate the dynamical onset of objectification for different values of the system frequency *ω*_*S*_. As an example, we plot in Fig. 1 the behaviour of *f*_5%_. The plot clearly shows that quantum Darwinism, indicated by small values of *f*_5%_ occurs very rapidly for values of the system frequency resonant with the peak of the spectral density while it is strongly inhibited in the off-resonant regime. The value *δ* = 5% is of course arbitrary, and the qualitative results do not depend on this particular choice; higher values would actually yield a wider flat central region (numerical results not shown), and lower values a narrower one. Furthermore choosing smaller *δ* is numerically disadvantageous since the algorithm has to run to bigger fragment sizes. The dependence of redundancy on resonance conditions is confirmed by the plots of Fig. 2 where we show the asymptotic mutual information as a function of *f* for resonant system frequency (red curve), at the edge of the spectral distribution (green curve) and for off-resonant frequency (blue curve). It is evident that the plateau disappears when the system frequency moves away from the peak of the spectrum. A note about initial conditions is in order: we could have chosen higher values of the squeezing parameter and this would have simply compressed all curves closer to the horizontal line, so we have chosen *r* = 3 so that the results are more clearly visible. We have also checked that by choosing an initial state delocalized in momentum (*r* < 0), decoherence happens half oscillator’s period later, as expected from former results^31^, highlighting the importance of the pointer observable (position; see^32^ for a more detailed analysis on pointers for this setup).

### Non-Markovianity and non-monotonic behaviour

**Figure 3:**
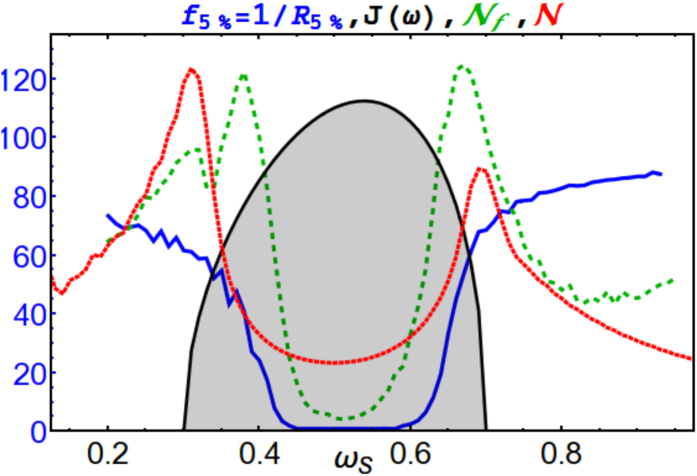
Spectral density *J*(*ω*) (shaded gray), non-Markovianity 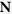 (red/dotted line), fraction *f*_*δ*_ = 1/*R*_*δ*_ (blue/solid line) with *δ* = 5%, and non-monotonicity of *f*_*δ*_ 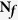 (green/dashed line) for *t* = 150, initial squeezing *r* = 10. Other parameters as in Fig. 1. All plots are in a.u.

In order to link this behaviour with memory effects arising from the structural features of the frequency spectrum we calculate the non-Markovianity measure 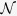 of equation (6) and compare its behaviour with the behaviour of the fraction *f*_5%_ when varying *ω*_*S*_. As one can see from Fig. 3, information flow back, and hence 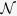, is maximal at the edge of the spectrum and minimal at the centre. This is consistent with the fact that on resonance the system energy is rapidly dissipated into the environment. As we move towards the edges of the distribution, the system oscillator is more strongly sensitive to the variations in the form of the spectrum and memory effects become dominant. When off-resonance, oscillatory behaviour dominates the dynamics. Small amplitude oscillations are also present in the redundancy (See Fig. 1) and in the dynamics of the mutual information 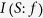.

We quantify this non-monotonic behaviour of *f*_*δ*_ by introducing its non-monotonicity defined in a similar fashion to 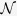, namely


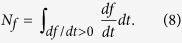


This quantity is plotted in Fig. 3 (green dashed line) and it shows a good qualitative agreement with the behaviour of the non-Markovianity measure 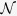. We have checked the validity of this result for different initial squeezings. For times relevant to quantum Darwinism, lower squeezings simply lead to worst plateaus, i.e. higher *f*_*δ*_. For completeness we note that for longer times, where dissipation begins to play a role, the shape in Fig. 3 gets more easily destroyed for lower squeezings; but of course, if we wait long enough we would also see thermalization where no trace of Darwinism is left.

There are hence three main regimes where we can highlight the connection between non-Markovianity and quantum Darwinism: when in resonance, the system dissipates monotonously into the bath, meaning a Markovian evolution and a constant tendency towards a perfect quantum Darwinism situation where stable redundant records are established in the environmental fractions. Out of resonance we have exactly the opposite situation, practically no dissipation and no bath’s information gain about the system (very poor Darwinism). However, near the frequency edges, information and energy flow back and forth from the system, meaning non-monotonic dissipation and non-Markovianity as well as non-monotonic 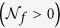 creation of records leading to poor Darwinism (in the sense of higher *f*_5%_). The very definition of record requires temporal stability, being this the main motivation to introduce the indicator 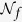.

### Analysis through decoherence factors

**Figure 4:**
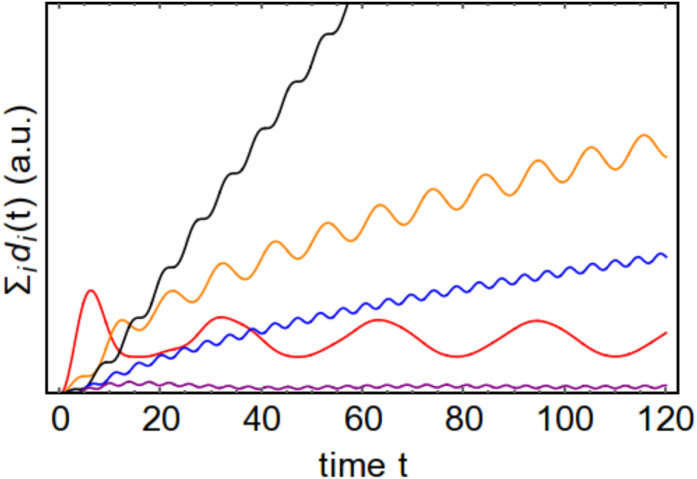
Sum of the decoherence factors 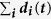 for *ω*_*S*_ = 0.1, 0.3, 0.5, 0.7, 0.8 corresponding to (red, orange, black, blue, purple). We have used the same parameters as those in Fig. 1.

All these cases can also be understood, according to the simple analytical model introduced in^26^, in terms of the decoherence factors *d*_*i*_(*t*) appearing in the system + fraction state (of spectrum-broadcast form) that we have discussed before. As argued there, each entropy appearing in the mutual information between system and a given fraction can be obtained from the symplectic areas of the corresponding collection of oscillators. The change in time of these areas is just the sum of decohering factors. For example, the change in symplectic area for the system oscillator is just the sum 
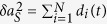
, while that of a fraction *F* is 
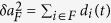
. Furthermore, it is argued^26^ that we can on average substitute 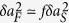, where *f* is the size of fraction *F*. This, together with the analytic expression for *d*_*i*_(*t*) allows to easily translate our intuition into a more particular representation: expressing the mutual information 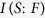 as a function of the fraction size *f* it is immediate to see that we need a high 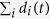 in order to have a plateau, and thus good Darwinism conditions. The analysis can be thus reduced to studying the evolution of the decoherence factors. We do so in Fig. 4, where it is seen that it grows in time to big values for the resonant case, meaning good redundancy and perfectly distinguishable records in each fragment. For the detuned cases it remains at low values as expected, yielding fuzzy records. In the edge cases it oscillates very often while increasing in a milder fashion. In the latter case we will have decent redundancy but with non-monotonic behaviour: records are formed and destroyed in a periodic fashion, although they ultimately improve with time.

This simple model reproduces remarkably well the predictions for quantum Darwinism^26^, as well as system + bath entanglement properties^27^, in this setup. At the same time, the decoherence rate of -position- off-diagonal elements of the system’s density matrix is also governed by the *d*_*i*_(*t*)^26^, meaning that their oscillatory behaviour will lead to recoherence, and thus non-Markovian, effects in the system’s evolution. We conclude that this is the mechanism underlying both non-Markovian evolution and the decreased ability of the bath to redundantly store classical information about the system.

## Discussion

We have studied the connection between non-Markovian dynamics, characterized by information flow back, and the emergence of an objective classical reality through the proliferation of classical copies of the system state in the normal modes of the environment. We have shown that, by modifying the properties of the spectral density, and, in particular, by changing the resonance condition between the system oscillator and the reservoir spectrum, we can inhibit quantum Darwinism by modifying the Markovian character of the dynamical map.

Evidence for such connection has been given both from a numerical perspective, where we have shown that three distinct regimes appear (resonant, far-detuned, edge) which are connected to well-known properties of the model related to its non-Markovian properties^28^ and to its ability to propagate information and energy^33^, and which are here shown to also be related to a hindering of quantum Darwinism. Furthermore we have used the model, which successfully explains Darwinism in this setup, introduced in^26^ and later used in^27^, to highlight that the three regimes can be understood from a unique underlying mechanism related to decohering factors, which can explain both non-Markovianity and quantum Darwinism. Finally, we comment on the possible generalization of our results. From the very description of decohering factors and the spectrum-broadcast form in eq. (5) it is easy to see that generalizing to non-Gaussian states will probably result in similar behaviour; indeed, fragments of the environment become more orthogonal for increasing decoherence factors, whichever the character of the system’s initial state (itself entering the expression of these factors as simple, innocuous, expected values in a multiplicative fashion), and thus good quantum Darwinism is expected to be achieved, although calculating entropic quantities in that regime (and the exact dynamics) would be more complicated.

It would also be of interest to consider further exploration of the found connection in other models to widen our understanding of its generality. Although it is hard to say which phenomenology awaits after each model, a recent result strengthening this connection has been found for a spin dissipating into a spin bath^34^. Also, a situation apparently close to our work would be a spin dissipating into a bosonic bath (the celebrated spin-boson model), maybe under some approximate regime as used here for the analytics. The quality of Darwinism in such scenario, together with unexplored effects of different environment states such as nonequilibrium situations, could be the subject of future research efforts.

## Methods

### Numerical simulation

We diagonalize the full (system + bath) problem, with *N* + 1 oscillators, and then obtain the relevant quantities. The mutual information for a given fraction size *f* is averaged over 100 different fractions of such size for each set of parameters (time and *ω*_*S*_). We have chosen a coupling strength 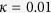. The non-Markovianity has been obtained similarly to^28^, calculating the time evolution of the fidelity between two different initial Gaussian states. Their phase difference runs along 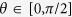 with Δ*θ* = *π*/4, while the squeezing parameters are *r*_1_ = 1 and 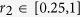 with Δ*r*_2_ = 0.25.

### Decoherence factors

The decoherence factor stemming from oscillator *i* (with frequency *ω*_*i*_ and mass *m*_*i*_) in the bath reads


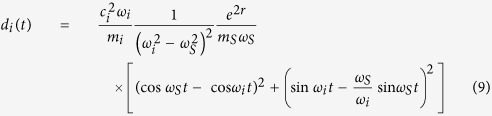


with *m*_*S*_ and *ω*_*S*_ the mass and frequency of the system, and *r* is the system squeezing parameter. An arbitrary spectral density *J*(*ω*) can be inserted into the problem by discretizing the bath’s frequency interval [*ω*_0_, *ω*_*R*_] into *N* oscillators as 
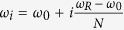
 and using the substitution


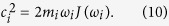


## Additional Information

**How to cite this article**: Galve, F. *et al.* Non-Markovianity hinders Quantum Darwinism. *Sci. Rep.* **6**, 19607; doi: 10.1038/srep19607 (2016).

## References

[b1] Zurek W. H. Quantum Darwinism. Nat. Phys. 5, 181 (2009).

[b2] Brandao F. G. S. L., Piani M. & Horodecki P. Generic emergence of classical features in quantum Darwinism. Nat. Comm. 6, 7908 (2015). 10.1038/ncomms8908 26264289

[b3] Haroche S. & Raimond J. M. Exploring the Quantum: Atoms, Cavities, and Photons (Oxford Graduate Texts, 2006).

[b4] Schlosshauer M. Decoherence, the measurement problem, and interpretations of quantum mechanics. Rev. Mod. Phys. 76, 1267 (2005).

[b5] Zurek W. H. Decoherence, einselection, and the quantum origins of the classical. Rev. Mod. Phys. 75, 715 (2003).

[b6] Wootters W. K. & Zurek W. H. A single quantum cannot be cloned. Nature 299, 802 (1982).

[b7] Blume-Kohout R. & Zurek W. H. A Simple Example of ‘Quantum Darwinism’: Redundant Information Storage in Many-Spin Environments. Found. Phys 35, 1857 (2004).

[b8] Zwolak M., Riedel C. J. & Zurek W. H. Amplification, Redundancy, and Quantum Chernoff Information. Phys. Rev. Lett. 112, 140406 (2014). 24765928 10.1103/PhysRevLett.112.140406

[b9] Riedel C. J. & Zurek W. H. Quantum Darwinism in an Everyday Environment: Huge Redundancy in Scattered Photons. Phys. Rev. Lett. 105, 020404 (2010). 20867689 10.1103/PhysRevLett.105.020404

[b10] Riedel C. J. & Zurek W. H. Redundant information from thermal illumination: quantum Darwinism in scattered photons. New J. Phys. 13, 073038 (2011).

[b11] Korbicz J. K., Horodecki P. & Horodecki R. Objectivity in a Noisy Photonic Environment through Quantum State Information Broadcasting. Phys. Rev. Lett. 112, 120402 (2014). 24724630 10.1103/PhysRevLett.112.120402

[b12] Horodecki R., Korbicz J. K. & Horodecki P. Quantum origins of objectivity. Phys. Rev. A 91, 032122 (2015).

[b13] Burke A. M. *et al.* Periodic Scarred States in Open Quantum Dots as Evidence of Quantum Darwinism. Phys. Rev. Lett. 104, 176801 (2010). 20482124 10.1103/PhysRevLett.104.176801

[b14] Breuer H. P., Laine E. M. & Piilo J. Measure for the Degree of Non-Markovian Behavior of Quantum Processes in Open Systems. Phys. Rev. Lett. 103, 210401 (2009). 20366019 10.1103/PhysRevLett.103.210401

[b15] Rivas A., Huelga S. F. & Plenio M. B. Entanglement and Non-Markovianity of Quantum Evolutions. Phys. Rev. Lett. 105, 050403 (2010). 20867898 10.1103/PhysRevLett.105.050403

[b16] Luo S., Fu S. & Song H. Quantifying non-Markovianity via correlations. Phys. Rev. A 86, 044101 (2012).

[b17] Lorenzo S., Plastina F. & Paternostro M. Geometrical characterization of non-Markovianity. Phys. Rev. A 88, 020102(R) (2013).

[b18] Chruściński D. & Maniscalco S. Degree of Non-Markovianity of Quantum Evolution. Phys. Rev. Lett. 112, 120404 (2014). 24724632 10.1103/PhysRevLett.112.120404

[b19] Vasile R., Olivares S., Paris M. G. A. & Maniscalco S. Continuous-variable quantum key distribution in non-Markovian channels. Phys. Rev. A 83, 042321 (2011).

[b20] Chin A. W., Huelga S. F. & Plenio M. B. Quantum Metrology in Non-Markovian Environments. Phys. Rev. Lett. 109, 233601 (2012). 23368199 10.1103/PhysRevLett.109.233601

[b21] Huelga S. F., Rivas A. & Plenio M. B. Non-Markovianity-Assisted Steady State Entanglement. Phys. Rev. Lett. 108, 160402 (2012). 22680702 10.1103/PhysRevLett.108.160402

[b22] Schmidt R., Negretti A., Ankerhold J., Calarco T. & Stockburger J. T. Optimal Control of Open Quantum Systems: Cooperative Effects of Driving and Dissipation. Phys. Rev. Lett. 107, 130404 (2011). 22026832 10.1103/PhysRevLett.107.130404

[b23] Xiang G. Y. *et al.* Entanglement distribution in optical fibers assisted by nonlocal memory effects. Eur. Phys. Lett. 107, 54006 (2014).

[b24] Laine E. M., Breuer H. P. & Piilo J. Nonlocal memory effects allow perfect teleportation with mixed states. Scient. Rep. 4, 4620 (2014). 10.1038/srep04620 PMC3980228 24714695

[b25] Bylicka B., Chruściński D. & Maniscalco S. Non-Markovianity and reservoir memory of quantum channels: a quantum information theory perspective. Scient. Rep. 4, 5720 (2014). 10.1038/srep05720 PMC4104480 25043763

[b26] Blume-Kohout R. & Zurek W. H. Quantum Darwinism in Quantum Brownian Motion. Phys. Rev. Lett. 101, 240405 (2008). 19113606 10.1103/PhysRevLett.101.240405

[b27] Paz J. P. & Roncaglia A. J. Redundancy of classical and quantum correlations during decoherence. Phys. Rev. A 80, 042111 (2009).

[b28] Vasile R., Galve F. & Zambrini R. Spectral origin of non-Markovian open-system dynamics: A finite harmonic model without approximations. Phys. Rev. A 89, 022109 (2014).

[b29] Rubin R. J. Momentum Autocorrelation Functions and Energy Transport in Harmonic Crystals Containing Isotopic Defects. Phys. Rev. 131, 964 (1963).

[b30] Vasile R., Maniscalco S., Paris M. G. A., Breuer H. P. & Piilo J. Quantifying non-Markovianity of continuous-variable Gaussian dynamical maps. Phys. Rev. A 84, 052118 (2011).

[b31] Paz J. P., Habib S. & Zurek W. H. Reduction of the wave packet: Preferred observable and decoherence time scale. Phys. Rev. D 47, 488 (1993). 10.1103/physrevd.47.488 10015604

[b32] Zurek W. H., Habib S. & Paz J. P. Coherent states via decoherence. Phys. Rev. Lett. 70, 1187 (1993). 10054313 10.1103/PhysRevLett.70.1187

[b33] Galve F. & Zambrini R. Energy and information propagation in a finite coupled bosonic heat bath. Int. J. Quant. Inf. 12, 1560022 (2014).

[b34] Giorgi G. L., Galve F. & Zambrini R. Quantum Darwinism and non-Markovian dissipative dynamics from quantum phases of the spin-1/2 XX model. Phys. Rev. A 92, 022105 (2015).

